# Ethyl 2-phenyl-3-(4-phenyl-1,2,3-selenadiazol-5-yl)-3-*p*-tolyl­propano­ate

**DOI:** 10.1107/S1600536811056017

**Published:** 2012-01-18

**Authors:** S. Sankari, P. Sugumar, T. Manisankar, S. Muthusubramanian, M. N. Ponnuswamy

**Affiliations:** a Department of Chemistry, Sri Sarada College for Women (Autonomus), Fairlands, Salem-600 016, India; b Centre of Advanced Study in Crystallography and Biophysics, University of Madras, Guindy Campus, Chennai 600 025, India; c Department of Industrial Chemistry, Alagappa University, Karaikudi 630 003, India; d School of Chemistry, Madurai Kamaraj University, Madurai 625 021, India

## Abstract

In the title compound, C_26_H_24_N_2_O_2_Se, the selenadiazole ring is essentially planar [maximum deviation = 0.004 (3) Å]. The dihedral angle between the selenadiazole ring and the attached benzene ring is 50.17 (1)°. The crystal packing is stabilized by inter­molecular C—H⋯N inter­actions.

## Related literature

For general background to selenadiazol derivatives, see: El-Bahaie *et al.* (1990 ▶); El-Kashef *et al.* (1986 ▶); Khanna (2005 ▶); Kuroda *et al.* (2001 ▶); Padmavathi *et al.* (2002 ▶); Plano *et al.* (2010 ▶); Stadtman (1991 ▶).
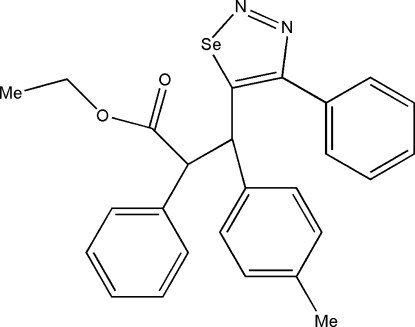


## Experimental

### 

#### Crystal data

C_26_H_24_N_2_O_2_Se
*M* *_r_* = 475.43
Monoclinic, 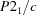

*a* = 12.148 (5) Å
*b* = 12.333 (5) Å
*c* = 16.522 (5) Å
β = 108.768 (5)°
*V* = 2343.7 (15) Å^3^
*Z* = 4
Mo *K*α radiation
μ = 1.63 mm^−1^
*T* = 293 K
0.20 × 0.20 × 0.15 mm

#### Data collection

Bruker SMART APEX CCD detector diffractometer
Absorption correction: multi-scan (*SADABS*; Bruker, 2008 ▶) *T*_min_ = 0.715, *T*_max_ = 0.784
21985 measured reflections
5739 independent reflections
3536 reflections with *I* > 2σ(*I*)
*R*_int_ = 0.038

#### Refinement

*R*[*F* ^2^ > 2σ(*F* ^2^)] = 0.042
*wR*(*F* ^2^) = 0.114
*S* = 1.02
5739 reflections
282 parameters
H-atom parameters constrained
Δρ_max_ = 0.63 e Å^−3^
Δρ_min_ = −0.30 e Å^−3^

### 

Data collection: *APEX2* (Bruker, 2008 ▶); cell refinement: *SAINT* (Bruker, 2008 ▶); data reduction: *SAINT*; program(s) used to solve structure: *SHELXS97* (Sheldrick, 2008 ▶); program(s) used to refine structure: *SHELXL97* (Sheldrick, 2008 ▶); molecular graphics: *ORTEP-3* (Farrugia, 1997 ▶); software used to prepare material for publication: *SHELXL97* and *PLATON* (Spek, 2009 ▶).

## Supplementary Material

Crystal structure: contains datablock(s) global, I. DOI: 10.1107/S1600536811056017/bt5750sup1.cif

Structure factors: contains datablock(s) I. DOI: 10.1107/S1600536811056017/bt5750Isup2.hkl

Supplementary material file. DOI: 10.1107/S1600536811056017/bt5750Isup3.cml

Additional supplementary materials:  crystallographic information; 3D view; checkCIF report

## Figures and Tables

**Table 1:** Hydrogen-bond geometry (Å, °)

*D*—H⋯*A*	*D*—H	H⋯*A*	*D*⋯*A*	*D*—H⋯*A*
C20—H20⋯N1^i^	0.93	2.61	3.522 (4)	165

Symmetry code: (i) 
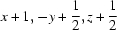
.

**Fig. 1.**
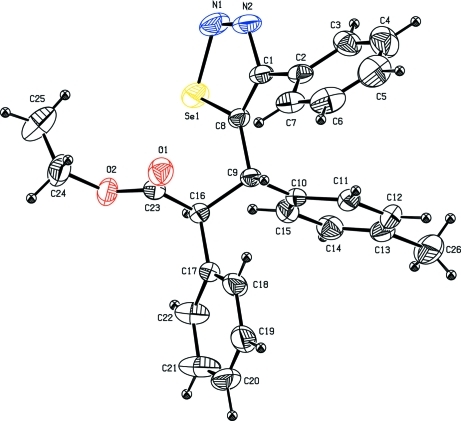
The molecular structure of the title compound, showing the atomic numbering and displacement ellipsoids drawn at the 30% probability level.

**Fig. 2.**
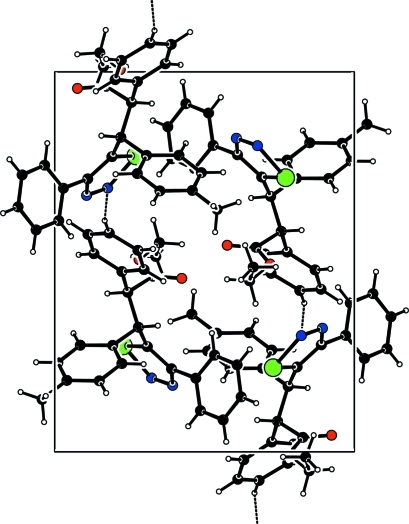
The packing of the molecules viewed down the b-axis.

## supplementary crystallographic information

### Comment

Selenadiazoles, having one selenium and two nitrogen atoms in a five membered ring, are the important class of organoselenium compounds utilized in the synthesis of semiconductor nanoparticles (Khanna, 2005). 1,2,3-Selenadiazoles are of interest owing to their chemical properties and biological applications such as anti-fungal (Kuroda *et al.*, 2001), anti-bacterial (El-Kashef *et al.*, 1986), anti-microbial (El-Bahaie *et al.*,1990), anti-cancer (Plano *et al.*, 2010) and insecticidal (Padmavathi *et al.*, 2002) properties.

Glutathione peroxidases(GPx) are the antioxidant selenoenzymes protecting various organisms from oxidative stress by catalyzing the reduction of hydroperoxides at the expense of glutathione(GSH) (Stadtman, 1991). In view of the above important properties of selenium containing compounds, an attempt is made to determine the crystal structure of the title compound.

The *ORTEP* plot of the molecule is shown in Fig.1. The selenadiazol ring is planar and oriented at an angle of 50.17 (1)° with the attached phenyl ring. The planar phenyl rings are twisted away across the propanyl bond at an angle of 73.7 (1)°. The propanoate group assumes partially an extended conformation which can seen from the torsion angle value of [C9—C16—C23—O2] 138.0 (2)°. The molecular packing is controlled by C—H···N intermolecular interactions in addition to van der Waals forces.

### Experimental

A mixture of ethyl-5-oxo-2,5-diphenyl-3-*p*-tolylpentanoate (1 mmol), semicarbazide hydrochloride(2 mmol) and anhydrous sodium acetate (3 mmol) in ethanol (10 ml) was refluxed for 4 hrs. After completion of the reaction as monitored by TLC, the mixture was poured into ice cold water and the resulting semicarbazone was filtered off. Then, a mixture of semicarbazone (1 mmol) and SeO_2_ (2 mmol) in tetrahydrofuran (10 ml) were refluxed on a water bath for 1hr. The selenium deposited on cooling was removed by filtration, and the filtrate was poured into crushed ice, extracted with dichloromethane, and purified by column chromatography using silica gel (60–120 mesh) with 97:3 petroleum ether: ethyl acetate as eluent to give ethyl-2-phenyl-3-(4-phenyl-1,2, 3-selenadiazol-5-yl)-3-*p*-tolylpropanoate.

### Refinement

H atoms were positioned geometrically (C—H = 0.93–0.98 Å) and allowed to ride on their parent atoms,with *U*_iso_(H) = 1.5*U*_eq_(C) for methyl H 1.2*U*_eq_(C) for other H atoms.

### Crystal data

**Table:**

C_26_H_24_N_2_O_2_Se	*F*(000) = 976
*M**_r_* = 475.43	*D*_x_ = 1.347 Mg m^−^^3^
Monoclinic, *P*2_1_/*c*	Mo *K*α radiation, λ = 0.71073 Å
Hall symbol: -P 2ybc	Cell parameters from 3536 reflections
*a* = 12.148 (5) Å	θ = 1.8–28.3°
*b* = 12.333 (5) Å	µ = 1.63 mm^−^^1^
*c* = 16.522 (5) Å	*T* = 293 K
β = 108.768 (5)°	Block, white crystalline
*V* = 2343.7 (15) Å^3^	0.20 × 0.20 × 0.15 mm
*Z* = 4	

### Data collection

**Table:**

Bruker SMART APEX CCD detector diffractometer	5739 independent reflections
Radiation source: fine-focus sealed tube	3536 reflections with *I* > 2σ(*I*)
graphite	*R*_int_ = 0.038
ω scans	θ_max_ = 28.3°, θ_min_ = 1.8°
Absorption correction: multi-scan (*SADABS*; Bruker, 2008)	*h* = −16→16
*T*_min_ = 0.715, *T*_max_ = 0.784	*k* = −16→15
21985 measured reflections	*l* = −22→21

### Refinement

**Table:**

Refinement on *F*^2^	Primary atom site location: structure-invariant direct methods
Least-squares matrix: full	Secondary atom site location: difference Fourier map
*R*[*F*^2^ > 2σ(*F*^2^)] = 0.042	Hydrogen site location: inferred from neighbouring sites
*wR*(*F*^2^) = 0.114	H-atom parameters constrained
*S* = 1.02	*w* = 1/[σ^2^(*F*_o_^2^) + (0.045*P*)^2^ + 0.8234*P*] where *P* = (*F*_o_^2^ + 2*F*_c_^2^)/3
5739 reflections	(Δ/σ)_max_ = 0.007
282 parameters	Δρ_max_ = 0.63 e Å^−^^3^
0 restraints	Δρ_min_ = −0.30 e Å^−^^3^

### Special details

**Table:**

Geometry. All e.s.d.'s (except the e.s.d. in the dihedral angle between two l.s. planes) are estimated using the full covariance matrix. The cell e.s.d.'s are taken into account individually in the estimation of e.s.d.'s in distances, angles and torsion angles; correlations between e.s.d.'s in cell parameters are only used when they are defined by crystal symmetry. An approximate (isotropic) treatment of cell e.s.d.'s is used for estimating e.s.d.'s involving l.s. planes.
Refinement. Refinement of *F*^2^ against ALL reflections. The weighted *R*-factor *wR* and goodness of fit *S* are based on *F*^2^, conventional *R*-factors *R* are based on *F*, with *F* set to zero for negative *F*^2^. The threshold expression of *F*^2^ > σ(*F*^2^) is used only for calculating *R*-factors(gt) *etc*. and is not relevant to the choice of reflections for refinement. *R*-factors based on *F*^2^ are statistically about twice as large as those based on *F*, and *R*- factors based on ALL data will be even larger.

### Fractional atomic coordinates and isotropic or equivalent isotropic displacement parameters (Å^2^)

**Table:**

	*x*	*y*	*z*	*U*_iso_*/*U*_eq_	
C1	0.6317 (3)	0.4864 (2)	0.10829 (19)	0.0747 (8)	
H1	0.5772	0.4457	0.0671	0.090*	
C2	0.6984 (4)	0.5621 (3)	0.0839 (3)	0.1018 (12)	
H2	0.6887	0.5724	0.0263	0.122*	
C3	0.7792 (4)	0.6224 (3)	0.1445 (3)	0.1074 (13)	
H3	0.8253	0.6723	0.1280	0.129*	
C4	0.7917 (3)	0.6089 (3)	0.2288 (3)	0.0909 (11)	
H4	0.8454	0.6510	0.2696	0.109*	
C5	0.7258 (2)	0.5336 (2)	0.25435 (19)	0.0663 (7)	
H5	0.7351	0.5248	0.3121	0.080*	
C6	0.6456 (2)	0.47105 (19)	0.19370 (16)	0.0548 (6)	
C7	0.57234 (19)	0.3898 (2)	0.21839 (15)	0.0511 (6)	
C8	0.60737 (18)	0.3091 (2)	0.27673 (14)	0.0466 (5)	
C9	0.73116 (18)	0.27921 (18)	0.32782 (13)	0.0432 (5)	
H9	0.7791	0.3442	0.3320	0.052*	
C10	0.77602 (18)	0.19359 (19)	0.28023 (13)	0.0444 (5)	
C11	0.8454 (2)	0.2243 (2)	0.23223 (16)	0.0542 (6)	
H11	0.8655	0.2969	0.2306	0.065*	
C12	0.8852 (2)	0.1489 (3)	0.18676 (17)	0.0687 (8)	
H12	0.9314	0.1716	0.1547	0.082*	
C13	0.8582 (2)	0.0407 (3)	0.18788 (17)	0.0673 (8)	
C14	0.7875 (3)	0.0105 (2)	0.23479 (17)	0.0674 (7)	
H14	0.7667	−0.0620	0.2356	0.081*	
C15	0.7469 (2)	0.0853 (2)	0.28051 (16)	0.0582 (6)	
H15	0.6996	0.0626	0.3118	0.070*	
C16	0.74223 (19)	0.24382 (19)	0.41975 (14)	0.0470 (5)	
H16	0.6991	0.1760	0.4166	0.056*	
C17	0.8681 (2)	0.2242 (2)	0.47442 (14)	0.0546 (6)	
C18	0.9536 (2)	0.2983 (3)	0.47948 (17)	0.0817 (10)	
H18	0.9356	0.3627	0.4488	0.098*	
C19	1.0676 (3)	0.2777 (4)	0.5303 (2)	0.1065 (14)	
H19	1.1259	0.3280	0.5337	0.128*	
C20	1.0928 (3)	0.1821 (4)	0.5755 (2)	0.1085 (14)	
H20	1.1690	0.1669	0.6085	0.130*	
C21	1.0084 (4)	0.1112 (4)	0.5723 (3)	0.1197 (15)	
H21	1.0255	0.0481	0.6047	0.144*	
C22	0.8967 (3)	0.1310 (3)	0.5213 (2)	0.0866 (10)	
H22	0.8391	0.0801	0.5185	0.104*	
C23	0.6893 (2)	0.3282 (3)	0.46202 (15)	0.0548 (6)	
C24	0.5688 (3)	0.3495 (3)	0.5502 (2)	0.0914 (10)	
H24A	0.5929	0.3296	0.6102	0.110*	
H24B	0.5898	0.4247	0.5462	0.110*	
C25	0.4454 (3)	0.3374 (3)	0.5139 (3)	0.1169 (14)	
H25A	0.4205	0.3645	0.4564	0.175*	
H25B	0.4078	0.3775	0.5473	0.175*	
H25C	0.4253	0.2621	0.5136	0.175*	
C26	0.9020 (3)	−0.0422 (3)	0.1379 (2)	0.1073 (13)	
H26A	0.8593	−0.0354	0.0781	0.161*	
H26B	0.8915	−0.1137	0.1571	0.161*	
H26C	0.9831	−0.0299	0.1468	0.161*	
N1	0.39102 (19)	0.3233 (3)	0.19430 (17)	0.0838 (8)	
N2	0.45350 (19)	0.3936 (2)	0.17484 (14)	0.0701 (6)	
O1	0.70126 (18)	0.42406 (18)	0.45645 (13)	0.0752 (5)	
O2	0.62809 (16)	0.28144 (16)	0.50584 (12)	0.0692 (5)	
Se1	0.48065 (2)	0.22922 (3)	0.27871 (2)	0.07445 (14)	

### Atomic displacement parameters (Å^2^)

**Table:**

	*U*^11^	*U*^22^	*U*^33^	*U*^12^	*U*^13^	*U*^23^
C1	0.090 (2)	0.0605 (18)	0.0701 (18)	0.0122 (16)	0.0208 (16)	0.0119 (14)
C2	0.136 (3)	0.083 (3)	0.098 (3)	0.019 (2)	0.053 (3)	0.029 (2)
C3	0.104 (3)	0.076 (3)	0.154 (4)	0.002 (2)	0.058 (3)	0.035 (3)
C4	0.071 (2)	0.059 (2)	0.131 (3)	−0.0017 (16)	0.017 (2)	0.007 (2)
C5	0.0551 (15)	0.0573 (17)	0.0774 (18)	0.0087 (13)	0.0085 (13)	0.0041 (14)
C6	0.0513 (13)	0.0466 (14)	0.0608 (15)	0.0148 (11)	0.0102 (11)	0.0049 (11)
C7	0.0396 (12)	0.0551 (15)	0.0512 (13)	0.0097 (10)	0.0045 (10)	−0.0050 (11)
C8	0.0366 (11)	0.0546 (14)	0.0452 (12)	0.0022 (10)	0.0083 (9)	−0.0033 (10)
C9	0.0352 (10)	0.0473 (13)	0.0442 (11)	0.0011 (9)	0.0085 (9)	0.0041 (10)
C10	0.0367 (11)	0.0522 (14)	0.0411 (11)	0.0048 (9)	0.0080 (9)	0.0061 (10)
C11	0.0448 (12)	0.0603 (15)	0.0583 (14)	0.0015 (11)	0.0176 (11)	0.0031 (12)
C12	0.0526 (15)	0.095 (2)	0.0645 (16)	0.0135 (15)	0.0268 (13)	0.0031 (15)
C13	0.0634 (16)	0.077 (2)	0.0576 (15)	0.0270 (15)	0.0138 (13)	0.0000 (14)
C14	0.0788 (18)	0.0554 (16)	0.0640 (16)	0.0120 (14)	0.0173 (15)	0.0007 (13)
C15	0.0626 (15)	0.0572 (16)	0.0564 (14)	0.0028 (12)	0.0215 (12)	0.0063 (12)
C16	0.0399 (11)	0.0549 (14)	0.0461 (12)	0.0030 (10)	0.0138 (9)	0.0046 (10)
C17	0.0457 (13)	0.0763 (17)	0.0405 (12)	0.0120 (12)	0.0120 (10)	0.0049 (12)
C18	0.0463 (15)	0.137 (3)	0.0567 (16)	−0.0064 (17)	0.0095 (12)	0.0272 (17)
C19	0.0470 (16)	0.210 (5)	0.0564 (18)	−0.013 (2)	0.0074 (14)	0.015 (2)
C20	0.060 (2)	0.183 (4)	0.066 (2)	0.043 (3)	−0.0019 (17)	−0.008 (3)
C21	0.101 (3)	0.109 (3)	0.108 (3)	0.041 (3)	−0.025 (2)	0.008 (2)
C22	0.078 (2)	0.075 (2)	0.083 (2)	0.0132 (17)	−0.0055 (16)	0.0092 (17)
C23	0.0440 (12)	0.0723 (19)	0.0446 (13)	0.0063 (12)	0.0093 (10)	0.0031 (12)
C24	0.076 (2)	0.126 (3)	0.085 (2)	0.001 (2)	0.0431 (17)	−0.025 (2)
C25	0.075 (2)	0.109 (3)	0.176 (4)	0.009 (2)	0.053 (3)	−0.033 (3)
C26	0.113 (3)	0.110 (3)	0.104 (3)	0.050 (2)	0.042 (2)	−0.015 (2)
N1	0.0359 (11)	0.112 (2)	0.0921 (18)	0.0028 (13)	0.0046 (12)	−0.0034 (16)
N2	0.0437 (12)	0.0825 (17)	0.0696 (14)	0.0147 (12)	−0.0020 (10)	0.0015 (12)
O1	0.0862 (14)	0.0675 (14)	0.0794 (13)	0.0092 (11)	0.0371 (11)	−0.0021 (10)
O2	0.0616 (11)	0.0898 (14)	0.0664 (11)	−0.0013 (10)	0.0349 (9)	−0.0087 (10)
Se1	0.04194 (15)	0.0938 (3)	0.0834 (2)	−0.01152 (14)	0.01441 (13)	0.00719 (16)

### Geometric parameters (Å, °)

**Table:**

C1—C2	1.379 (5)	C15—H15	0.9300
C1—C6	1.379 (4)	C16—C23	1.508 (4)
C1—H1	0.9300	C16—C17	1.525 (3)
C2—C3	1.373 (6)	C16—H16	0.9800
C2—H2	0.9300	C17—C18	1.365 (4)
C3—C4	1.361 (5)	C17—C22	1.367 (4)
C3—H3	0.9300	C18—C19	1.392 (4)
C4—C5	1.379 (4)	C18—H18	0.9300
C4—H4	0.9300	C19—C20	1.376 (6)
C5—C6	1.385 (4)	C19—H19	0.9300
C5—H5	0.9300	C20—C21	1.336 (6)
C6—C7	1.482 (4)	C20—H20	0.9300
C7—C8	1.355 (3)	C21—C22	1.369 (5)
C7—N2	1.391 (3)	C21—H21	0.9300
C8—C9	1.514 (3)	C22—H22	0.9300
C8—Se1	1.837 (2)	C23—O1	1.198 (3)
C9—C10	1.518 (3)	C23—O2	1.325 (3)
C9—C16	1.544 (3)	C24—C25	1.432 (5)
C9—H9	0.9800	C24—O2	1.449 (3)
C10—C15	1.383 (3)	C24—H24A	0.9700
C10—C11	1.383 (3)	C24—H24B	0.9700
C11—C12	1.377 (4)	C25—H25A	0.9600
C11—H11	0.9300	C25—H25B	0.9600
C12—C13	1.376 (4)	C25—H25C	0.9600
C12—H12	0.9300	C26—H26A	0.9600
C13—C14	1.381 (4)	C26—H26B	0.9600
C13—C26	1.514 (4)	C26—H26C	0.9600
C14—C15	1.380 (4)	N1—N2	1.261 (3)
C14—H14	0.9300	N1—Se1	1.870 (3)
			
C2—C1—C6	120.1 (3)	C23—C16—C9	110.20 (19)
C2—C1—H1	119.9	C17—C16—C9	112.39 (18)
C6—C1—H1	119.9	C23—C16—H16	108.3
C3—C2—C1	120.2 (4)	C17—C16—H16	108.3
C3—C2—H2	119.9	C9—C16—H16	108.3
C1—C2—H2	119.9	C18—C17—C22	118.6 (3)
C4—C3—C2	119.9 (4)	C18—C17—C16	121.9 (2)
C4—C3—H3	120.0	C22—C17—C16	119.5 (2)
C2—C3—H3	120.0	C17—C18—C19	120.3 (3)
C3—C4—C5	120.7 (3)	C17—C18—H18	119.9
C3—C4—H4	119.7	C19—C18—H18	119.9
C5—C4—H4	119.7	C20—C19—C18	119.2 (4)
C4—C5—C6	119.7 (3)	C20—C19—H19	120.4
C4—C5—H5	120.1	C18—C19—H19	120.4
C6—C5—H5	120.1	C21—C20—C19	120.3 (3)
C1—C6—C5	119.3 (3)	C21—C20—H20	119.8
C1—C6—C7	119.2 (2)	C19—C20—H20	119.8
C5—C6—C7	121.5 (2)	C20—C21—C22	120.3 (4)
C8—C7—N2	115.4 (2)	C20—C21—H21	119.9
C8—C7—C6	127.8 (2)	C22—C21—H21	119.9
N2—C7—C6	116.7 (2)	C17—C22—C21	121.3 (4)
C7—C8—C9	127.1 (2)	C17—C22—H22	119.4
C7—C8—Se1	109.47 (16)	C21—C22—H22	119.4
C9—C8—Se1	123.15 (17)	O1—C23—O2	125.1 (2)
C8—C9—C10	109.78 (17)	O1—C23—C16	124.4 (2)
C8—C9—C16	111.95 (18)	O2—C23—C16	110.5 (2)
C10—C9—C16	112.37 (18)	C25—C24—O2	110.6 (3)
C8—C9—H9	107.5	C25—C24—H24A	109.5
C10—C9—H9	107.5	O2—C24—H24A	109.5
C16—C9—H9	107.5	C25—C24—H24B	109.5
C15—C10—C11	118.0 (2)	O2—C24—H24B	109.5
C15—C10—C9	122.4 (2)	H24A—C24—H24B	108.1
C11—C10—C9	119.5 (2)	C24—C25—H25A	109.5
C12—C11—C10	120.9 (3)	C24—C25—H25B	109.5
C12—C11—H11	119.5	H25A—C25—H25B	109.5
C10—C11—H11	119.5	C24—C25—H25C	109.5
C13—C12—C11	121.3 (3)	H25A—C25—H25C	109.5
C13—C12—H12	119.3	H25B—C25—H25C	109.5
C11—C12—H12	119.3	C13—C26—H26A	109.5
C12—C13—C14	117.7 (3)	C13—C26—H26B	109.5
C12—C13—C26	121.3 (3)	H26A—C26—H26B	109.5
C14—C13—C26	121.0 (3)	C13—C26—H26C	109.5
C15—C14—C13	121.5 (3)	H26A—C26—H26C	109.5
C15—C14—H14	119.2	H26B—C26—H26C	109.5
C13—C14—H14	119.2	N2—N1—Se1	111.30 (17)
C14—C15—C10	120.5 (2)	N1—N2—C7	117.0 (2)
C14—C15—H15	119.7	C23—O2—C24	118.8 (2)
C10—C15—H15	119.7	C8—Se1—N1	86.80 (11)
C23—C16—C17	109.34 (19)		
			
C6—C1—C2—C3	0.1 (5)	C11—C10—C15—C14	0.7 (3)
C1—C2—C3—C4	−1.4 (6)	C9—C10—C15—C14	178.5 (2)
C2—C3—C4—C5	1.4 (6)	C8—C9—C16—C23	−52.2 (3)
C3—C4—C5—C6	−0.2 (5)	C10—C9—C16—C23	−176.23 (19)
C2—C1—C6—C5	1.1 (4)	C8—C9—C16—C17	−174.4 (2)
C2—C1—C6—C7	179.6 (3)	C10—C9—C16—C17	61.6 (3)
C4—C5—C6—C1	−1.0 (4)	C23—C16—C17—C18	−72.9 (3)
C4—C5—C6—C7	−179.5 (2)	C9—C16—C17—C18	49.8 (3)
C1—C6—C7—C8	129.3 (3)	C23—C16—C17—C22	105.8 (3)
C5—C6—C7—C8	−52.2 (4)	C9—C16—C17—C22	−131.5 (3)
C1—C6—C7—N2	−48.3 (3)	C22—C17—C18—C19	1.1 (4)
C5—C6—C7—N2	130.1 (2)	C16—C17—C18—C19	179.8 (3)
N2—C7—C8—C9	174.4 (2)	C17—C18—C19—C20	−0.2 (5)
C6—C7—C8—C9	−3.3 (4)	C18—C19—C20—C21	−1.6 (6)
N2—C7—C8—Se1	−0.1 (3)	C19—C20—C21—C22	2.5 (7)
C6—C7—C8—Se1	−177.8 (2)	C18—C17—C22—C21	−0.2 (5)
C7—C8—C9—C10	−91.6 (3)	C16—C17—C22—C21	−178.9 (3)
Se1—C8—C9—C10	82.2 (2)	C20—C21—C22—C17	−1.6 (6)
C7—C8—C9—C16	142.9 (2)	C17—C16—C23—O1	82.0 (3)
Se1—C8—C9—C16	−43.3 (3)	C9—C16—C23—O1	−42.0 (3)
C8—C9—C10—C15	−78.9 (3)	C17—C16—C23—O2	−98.0 (2)
C16—C9—C10—C15	46.4 (3)	C9—C16—C23—O2	138.0 (2)
C8—C9—C10—C11	98.8 (2)	Se1—N1—N2—C7	−0.7 (3)
C16—C9—C10—C11	−135.9 (2)	C8—C7—N2—N1	0.6 (3)
C15—C10—C11—C12	−0.6 (3)	C6—C7—N2—N1	178.5 (2)
C9—C10—C11—C12	−178.5 (2)	O1—C23—O2—C24	0.9 (4)
C10—C11—C12—C13	−0.4 (4)	C16—C23—O2—C24	−179.1 (2)
C11—C12—C13—C14	1.4 (4)	C25—C24—O2—C23	114.7 (3)
C11—C12—C13—C26	−180.0 (3)	C7—C8—Se1—N1	−0.20 (19)
C12—C13—C14—C15	−1.3 (4)	C9—C8—Se1—N1	−175.0 (2)
C26—C13—C14—C15	−180.0 (3)	N2—N1—Se1—C8	0.5 (2)
C13—C14—C15—C10	0.3 (4)		

### Hydrogen-bond geometry (Å, °)

**Table:**

*D*—H···*A*	*D*—H	H···*A*	*D*···*A*	*D*—H···*A*
C20—H20···N1^i^	0.93	2.61	3.522 (4)	165

Symmetry codes: (i) *x*+1, −*y*+1/2, *z*+1/2.
